# Universal and Serial Laboratory Testing for SARS-CoV-2 at a Long-Term Care Skilled Nursing Facility for Veterans — Los Angeles, California, 2020

**DOI:** 10.15585/mmwr.mm6921e1

**Published:** 2020-05-29

**Authors:** Amy V. Dora, Alexander Winnett, Lauren P. Jatt, Kusha Davar, Mika Watanabe, Linda Sohn, Hannah S. Kern, Christopher J. Graber, Matthew B. Goetz

**Affiliations:** ^1^Division of Infectious Diseases, University of California, Los Angeles; ^2^David Geffen School of Medicine, University of California, Los Angeles; ^3^Division of Geriatrics and Extended Care, Veterans Affairs Greater Los Angeles Healthcare System, Los Angeles, California; ^4^Division of Infectious Diseases, Veterans Affairs Greater Los Angeles Healthcare System, Los Angeles, California.

On March 28, 2020, two residents of a long-term care skilled nursing facility (SNF) at the Veterans Affairs Greater Los Angeles Healthcare System (VAGLAHS) had positive test results for SARS-CoV-2, the cause of coronavirus disease 2019 (COVID-19), by reverse transcription–polymerase chain reaction (RT-PCR) testing of nasopharyngeal specimens collected on March 26 and March 27. During March 29–April 23, all SNF residents, regardless of symptoms, underwent serial (approximately weekly) nasopharyngeal SARS-CoV-2 RT-PCR testing, and positive results were communicated to the county health department. All SNF clinical and nonclinical staff members were also screened for SARS-CoV-2 by RT-PCR during March 29–April 10. Nineteen of 99 (19%) residents and eight of 136 (6%) staff members had positive test results for SARS-CoV-2 during March 28–April 10; no further resident cases were identified on subsequent testing on April 13, April 22, and April 23. Fourteen of the 19 residents with COVID-19 were asymptomatic at the time of testing. Among these residents, eight developed symptoms 1–5 days after specimen collection and were later classified as presymptomatic; one of these patients died. This report describes an outbreak of COVID-19 in an SNF, with case identification accomplished by implementing several rounds of RT-PCR testing, permitting rapid isolation of both symptomatic and asymptomatic residents with COVID-19. The outbreak was successfully contained following implementation of this strategy.

VAGLAHS includes 150 long-term care beds in three SNF patient care areas, or wards; SNF wards A and B are in building 1, and ward C is in building 2. Buildings 1 and 2 do not share common areas, but residents might have indirect contact with outside persons while receiving medical services such as dialysis. These wards admit residents who require intravenous antibiotics, complex wound care, other rehabilitation needs, routine dialysis, chemotherapy, or radiation therapy; underlying conditions, including chronic obstructive pulmonary disease, hypertension, cardiovascular disease, and chronic kidney disease, are common. At the time of the outbreak, 99 (66%) beds were occupied; >95% of residents were men aged 50–100 years. All data were abstracted from the VAGLAHS electronic health record system on which all records are maintained on inpatients, SNF residents, and outpatients. 

To reduce the risk for introduction of SARS-CoV-2, on March 6, all VAGLAHS staff members and visitors were screened for symptoms of COVID-19 (i.e., fever, cough, or shortness of breath), travel to countries that had CDC travel warnings for COVID-19, and any close contact with persons with known COVID-19; those with relevant symptoms or exposures were not allowed entry to any area of the facility. On March 11, all SNF admissions were suspended, and daily temperature and symptom screening began for all residents. Residents with fever or lower respiratory tract signs or symptoms were placed on droplet and contact precautions in single-person rooms. On March 17, visitors were prohibited from entering any SNF building.

On March 26, the index patient (patient A0.1^†^) in ward A developed fever. A second ward A patient (patient A0.2) developed fever and cough on March 27. Nasopharyngeal swabs collected the day of fever onset were reported as positive for SARS-CoV-2 for both patients A0.1 and A0.2 on March 28. In response, during March 29–31, VAGLAHS staff members screened all building 1 (wards A and B) residents, regardless of symptoms, by SARS-CoV-2 RT-PCR testing of nasopharyngeal swabs. On March 29, a resident from ward C (C0.1) in building 2 became symptomatic; SARS-CoV-2 RT-PCR nasopharyngeal testing was positive on March 30, prompting testing of all building 2 residents on March 31. All three residents with a diagnosis of COVID-19 (patients A0.1, A0.2, and C0.1) were transferred to the affiliated acute care hospital for isolation and clinical management.

Implementation of infection control procedures (i.e., hand hygiene, droplet and contact precautions for persons with fever or lower respiratory tract signs or symptoms), and strategies for case identification and containment were reviewed with SNF staff members. Although staff members could previously be assigned to daily shifts on different wards, beginning on March 28, each staff member was assigned to a single ward. During the outbreak, an infection control nurse regularly reviewed and monitored the use of recommended personal protective equipment (PPE) with all SNF staff members. Protocols for use of PPE, based on CDC guidance,^§^ did not change during the outbreak. All staff members were screened by RT-PCR at least once during March 29–April 10.

## RT-PCR Testing of Residents

RT-PCR testing of all residents, conducted during March 29–March 31 in wards A, B, and C, identified SARS-CoV-2 in four (13%) of 30 residents on ward A, none of 30 residents on ward B, and 10 (28%) of 36 residents on ward C. All infected residents were transferred to the affiliated hospital for isolation and clinical management, and the wards were closed to new admissions. Following the initial testing, some residents moved between the SNF and the affiliated hospital for treatment of medical conditions unrelated to COVID-19.

Considering the number of cases identified through initial testing, the Infection Control team, in coordination with the SNF nursing staff members, implemented serial (approximately weekly) RT-PCR testing among residents of wards A and C until no additional residents received a positive test result. On April 3, all 22 remaining ward A residents received negative test results and were subsequently transferred to wards B and C. Ward A was converted into a COVID-19 recovery unit to cohort patients without acute hospital needs with continued RT-PCR–positive test results during convalescence. On April 6, the 28 residents on ward C were retested; two had positive test results and were transferred to the COVID-19 recovery unit (Box). A third round of testing was performed on ward C on April 13; all 27 residents had negative test results. During April 22–23, a final round of testing conducted on wards B and C identified no positive test results among the remaining 83 residents.

BOX **Discharge criteria for Veterans Affairs Greater Los Angeles Healthcare System (VAGLAHS) facility patients with positive test results for SARS-CoV-2 and criteria for transfer back to acute care hospital — Los Angeles, California, 2020**

**Required criteria for discharge from acute care to COVID-19 recovery unit** *

- Confirmed COVID-19 diagnosis
- During the preceding 2 days
  - Temperature <100°F (<37.8°C)
  - Respiratory rate <24 per minute
- The day before discharge
  - Room air pulse oximetry >93% or no change from established baseline for residents with chronic oxygen requirement for 24 hours before transfer
  - D-dimer <2 *μ*g/mL FEU (per VAGLAHS test readout) within 24 hours before transfer
  - White blood cells <11,000/*μ*L
- Resident satisfies all other eligibility criteria for admission to VA SNF

**Required criteria for discharge from COVID-19 recovery unit to VA SNF** **^†^**

- 14 days have passed since admission to hospital and no fever for ≥72 hours without the use of fever-reducing medications and
- Negative results of a Food and Drug Administration Emergency Use Authorized COVID-19 molecular assay for detection of SARS-CoV-2 RNA from at least two consecutive nasopharyngeal swab specimens collected ≥24 hours apart (total of two negative specimens)

**Required criteria for transfer back to acute care hospital**

- Room air pulse oximetry <94% or change from established baseline for residents with chronic oxygen requirement
- Signs or symptoms as per the judgment of the COVID-19 recovery unit staff members
- Within a 24-hour period, both of the following:
  - Temperature >99.9°F (>37.7°C)
  - Respiratory rate ≥24 per minute

**Abbreviations:** COVID-19 = coronavirus disease 2019; FEU = fibrinogen equivalent units; SNF = long-term care skilled nursing facility; VA = Veterans Affairs.

* Laboratory tests are not required for asymptomatic comfort care residents who are otherwise candidates for transfer to the COVID-19 recovery unit.

^†^ A test-based strategy is preferred for discontinuation of transmission-based precautions for residents who are being transferred to a long-term care or assisted living facility. All testing must be complete before transfer.

In total, three residents were identified with COVID-19 based on testing conducted because of symptoms, and 16 additional residents were identified with COVID-19 because of RT-PCR testing, two of whom reported or were identified with symptoms at the time of RT-PCR testing (Table). Fourteen of the 19 (74%) residents with COVID-19 reported no symptoms at the time of testing; among these residents, eight were presymptomatic, developing symptoms 1–5 days after the date of specimen collection. One of the three initially identified patients, C0.1, a man aged >90 years, died.

**TABLE:** Characteristics of long-term care skilled nursing facility residents with positive test results for SARS-CoV-2 (N = 19) — Veterans Affairs Greater Los Angeles Healthcare System, Los Angeles, California, 2020

Characteristic	No. (%)			
	Asymptomatic* (n = 6)	Presymptomatic* (n = 8)	Symptomatic* (n = 5)	All (N = 19)
**Demographic**				
**Age, yrs, median (IQR)**	75 (72–75)	67 (66–84.5)	84 (70–85)	75 (66–85)
**Male sex**	6 (100)	8 (100)	5 (100)	19 (100)
**Race/Ethnicity^†^**				
Asian	—	—	—	—
Black or African American	2 (33)	4 (50)	2 (40)	8 (42)
Native Hawaiian or Pacific Islander	—	1 (13)	—	1 (5)
White	3 (50)	3 (38)	2 (40)	8 (42)
Unknown	1 (17)	—	1 (20)	2 (11)
Hispanic	—	—	—	—
**Underlying medical condition^§^**				
Hypertension	5 (83)	5 (63)	3 (60)	13 (68)
Cardiovascular disease	3 (50)	4 (50)	5 (100)	12 (63)
Diabetes	4 (67)	5 (63)	2 (40)	11 (58)
Body mass index >30 kg/m^2^	3 (50)	2 (25)	2 (40)	7 (37)
Chronic kidney disease (stage 4 or above)	—	2 (25)	1 (20)	3 (16)
Chronic obstructive pulmonary disease	1 (17)	1 (13)	2 (40)	4 (21)
**Symptoms at time of or after testing^¶^**				
**Constitutional symptom**	—	6 (75)	5 (100)	11 (58)
Fever	—	6 (75)	5 (100)	11 (58)
Myalgia	—	—	1 (20)	1 (5)
Headache	—	1 (13)	1 (20)	2 (11)
**Respiratory symptom**	—	4 (38)	5 (100)	9 (47)
Cough	—	2 (25)	5 (100)	7 (37)
Dyspnea	—	2 (25)	1 (20)	3 (16)
**Gastrointestinal symptom**	—	5 (63)	1 (20)	6 (32)
Nausea	—	1 (13)	—	1 (5)
Emesis	—	1 (13)	—	1 (5)
Diarrhea	—	2 (25)	—	2 (11)
Poor appetite	—	3 (38)	1 (20)	4 (21)
Laboratory findings on admission,**^,††^ median (IQR) [No.]				
WBC (x 1,000/*μ*L)	4.32 (3.67–5.91) [5]	4.35 (3.93–6.10) [8]	6.24 (6.09–7.08) [5]	5.32 (3.94–6.20) [18]
Lymphocytes (%)	31.5 (26.4–32.7) [5]	22.0 (17.5–25.9) [8]	16.7 (11.4–16.9) [5]	22.0 (17.0–30.3) [18]
Lymphocytes (x 1,000/*μ*L)	1,200 (1,140–1,200) [5]	960 (775–1,105) [8]	880 (770–1,200) [5]	1,025 (835–1,200) [18]
Creatinine (mg/dL)	1.00 (0.89–1.05) [4]	1.01 (0.82–1.07) [8]	2.84 (1.99–3.23) [5]	1.04 (0.88–1.41) [17]
AST (U/L)	19 (17–21) [3]	24 (20–29) [5]	31 (NA) [1]	22 (19–29) [9]
ALT (U/L)	16 (13–21) [4]	17 (14–44) [6]	28 (21–28) [3]	16 (14–28) [13]
D–Dimer (*μ*g/mL FEU)	0.54 (0.42–0.83) [4]	0.66 (0.55–1.42) [7]	0.94 (0.59–1.17) [3]	0.63 (0.50–1.29) [14]
Ferritin (ng/mL)	60.8 (51.2–99.7) [5]	343.0 (162.5–540.6) [8]	184.6 (NA) [2]	179.1 (59.0–354.2) [15]
CRP (mg/dL)	0.605 (0.420–1.190) [4]	1.070 (0.900–2.565) [7]	6.765 (NA) [2]	1.03 (0.71–2.63) [13]
**Outcomes**				
Supplemental oxygen required	—	4 (50)	4 (80)	8 (42)
Death	—	—	1 (20)	1 (5)
Length of hospital stay, days, median (IQR)	6 (1–6)	9 (7–10)	10 (5–13)	6 (5–10)

**Abbreviations**: ALT = alanine aminotransferase; AST = aspartate aminotransferase; CRP = C-reactive protein; FEU = fibrinogen equivalent units; IQR = interquartile range (1st–3rd); NA = not applicable; WBC = white blood cell.

* Patients were classified as symptomatic if they had at least one listed symptom at the time of first positive specimen collection, presymptomatic if they did not exhibit symptoms at the time of specimen collection but later developed at least one listed symptom, and asymptomatic if they did not exhibit symptoms at any time between specimen collection and the last date of data collection.

^†^ Asian, black, Native Hawaiian or Pacific Islander, and white residents in this cohort were non-Hispanic; Hispanic persons could be of any race.

^§^ Comorbidities were determined based on documented SNOMED CT and *International Classification of Diseases, Ninth Revision* codes and review of patient’s vital signs, laboratory values, imaging findings, and provider notes. Chronic kidney disease stage was calculated using the Cockcroft-Gault equation to determine creatinine clearance; patients with estimated glomerular filtration rates <30 mL per minute were considered stage 4 and above. One symptomatic patient was dialysis-dependent. Cardiovascular disease includes coronary artery disease, peripheral artery disease, and previous cerebrovascular accident.

^¶^ Symptoms were collected through review of all provider notes from March 26 through April 20. Constitutional, respiratory, and digestive symptoms were counted if any one of the symptoms at the time of or after testing was present as a change from baseline. Fever includes measured temperature >100.4°F (>38°C) or fever reported by provider.

** These values include the first available laboratory results within 48 hours of admission for each patient.

^††^ Reference values are as follows: WBC = 4.5–11.0 x 1,000 per *μ*L; lymphocytes = 600–4,800 x 1,000 per *μ*L; % lymphocytes = 20%–40%; creatinine = 0.66–1.28 mg per dL; AST = 13–35 U per liter; ALT = 7–45 U per liter; d-Dimer = 0.00–0.42 *μ*g per mL FEU; ferritin = 22–322 ng per mL; CRP = 0–0.744 mg per dL.

## RT-PCR Testing of Staff Members

During March 29–April 10, universal RT-PCR testing of all 136 staff members identified eight (6%) infections: three in registered nurses and five in licensed vocational nurses, all of whom worked in wards A or C. Four of the eight infected staff members were symptomatic and were tested within 2 days after symptom onset; one developed fever at work and was immediately tested and sent home. None of the others worked during or after symptom onset. Although serial RT-PCR testing of staff members was not feasible because of limited testing supplies, testing remained available for symptomatic staff members. No cases among staff members were identified after the initial round of testing.

## Discussion

During March 26–April 23, a total of 19 cases of COVID-19 were diagnosed among 99 SNF residents (19.2%). At the time of diagnosis, 14 of 19 residents were asymptomatic, eight of whom were presymptomatic; one patient died. One half of the eight staff members with a diagnosis of COVID-19 were initially asymptomatic. This report demonstrates the high prevalence of asymptomatic SARS-CoV-2 infection that can occur in SNFs, highlighting the potential for widespread transmission among residents and staff members before illness is recognized and demonstrating the utility of universal RT-PCR testing for COVID-19 after case identification in this setting.

SNFs and other long-term care facilities where residents have high rates of underlying medical conditions are particularly susceptible to COVID-19 outbreaks (*1*–*3*). Limited testing and delayed recognition of symptomatic cases in congregate living settings can result in large and protracted outbreaks (*3*). In a recently described outbreak within homeless shelters, RT-PCR testing of all residents, coupled with rapid isolation and cohorting procedures, limited transmission (*4*).

Multiple studies have demonstrated efficient transmission of SARS-CoV-2 from infected persons who are not yet symptomatic (*1*,*5*,*6*). One study in Italy showed through community surveillance testing that 43% of persons with confirmed SARS-CoV-2 infection were asymptomatic and that transmission from asymptomatic and presymptomatic persons also occurred within households.^¶^ In this cohort, transmission from asymptomatic persons was likely, because a large proportion of residents and staff members did not have symptoms at the time of diagnosis.

RT-PCR testing among SNF residents was repeated approximately weekly until all residents had negative test results. Serial testing aided the identification of subsequent cases. Testing of staff members might be especially important because they can acquire SARS-CoV-2 in the community and reintroduce it into the SNF. Although serial laboratory testing of staff members was considered after the initial round of testing, insufficient supplies limited the ability to fully carry this out.

Swift isolation and cohorting of residents with COVID-19 reduced further transmission within the SNF; residents who had positive test results were quickly transferred out of the SNF, either to the acute care hospital or directly to a separate COVID-19 recovery unit. The conversion of ward A into a COVID-19 recovery unit allowed cohorting of clinically stable residents within the SNF without requiring transfer to the affiliated hospital. This measure decreased burden on the hospital and allowed residents to remain in a familiar setting. Restricting staff movement between SNF wards reduced potential for transmission between wards. With these measures, the outbreak in ward A was suppressed within 1 week, the outbreak in ward C was suppressed within 2 weeks, and no cases occurred in ward B.

The Centers for Medicare & Medicaid Services currently recommends symptom screening of all SNF patients and cohorting of staffing teams for infected and uninfected patients (*7*). Medicare has expanded coverage for SARS-CoV-2 tests (*7*), and, as of April 30, Los Angeles County Department of Public Health had endorsed mass testing if a COVID-19 case is identified in a long-term care facility (*8*). At the time of the VAGLAHS SNF outbreak, the Los Angeles County Department of Public Health criteria for testing did not include RT-PCR testing of asymptomatic persons (*9*).

The findings in this report are subject to at least three limitations. First, because residents’ recall might be limited by cognitive disorders or recall bias, over- or underreporting of symptoms was possible and could have affected classification of patients as symptomatic or asymptomatic. Second, symptom data obtained from medical records might have been incomplete, because the daily symptom screening only included fever and respiratory symptoms and did not include symptoms more recently recognized as being associated with COVID-19, such as loss of sense of smell or taste,** which could have led to an overestimation of the asymptomatic population. Finally, because the all-male cohort of patients with laboratory-confirmed COVID-19 might have comorbidity profiles that differ from other groups, these findings might not be generalizable to other SNFs.

This investigation demonstrates the benefit of RT-PCR testing of SNF residents and staff members for SARS-CoV-2 after an initial case of COVID-19 is diagnosed. Identification of asymptomatic COVID-19 cases after initial RT-PCR testing supports implementation of serial laboratory testing in SNFs where COVID-19 cases have been identified. Identification of asymptomatic and presymptomatic residents with positive laboratory results for SARS-CoV-2 facilitated rapid transfer of these residents out of the SNF until a dedicated ward to cohort those with COVID-19 was created within the SNF, thereby reducing transmission. In congregate living settings that include persons with conditions that might place them at high risk for severe COVID-19, universal and serial laboratory-based testing for SARS-CoV-2 is an effective strategy that can be implemented for rapid identification of infection to minimize transmission.

**Summary**

**What is already known about this topic?**

Long-term care skilled nursing facilities (SNFs) are at high risk for COVID-19 outbreaks. Many SNF residents and staff members identified with COVID-19 are asymptomatic and presymptomatic.

**What is added by this report?**

After identification of two cases of COVID-19 in an SNF in Los Angeles, universal, serial reverse transcription–polymerase chain reaction (RT-PCR) testing of residents and staff members aided in rapid identification of additional cases and isolation and cohorting of these residents and interruption of transmission in the facility.

**What are the implications for public health practice?**

Universal and serial RT-PCR testing in SNFs can identify cases during an outbreak, and rapid isolation and cohorting can help interrupt transmission.

## Footnotes

* These authors contributed equally to this report.

† Residents in this report are labeled as follows: the first character (A, B, C) represents the originating ward of the patient with a diagnosis of COVID-19; the numeric character preceding the decimal point represents whether they were identified as an index patient (0) or in a round of surveillance testing (1, 2); the numeric character following the decimal point (1–10) represents the individual patient ordered chronologically by receipt of positive test result.

§ https://www.cdc.gov/coronavirus/2019-ncov/hcp/infection-control.html.

¶ https://www.medrxiv.org/content/10.1101/2020.04.17.20053157v1.

** https://www.cdc.gov/coronavirus/2019-ncov/hcp/clinical-criteria.html.
